# Fatigue Resistance and Cracking Mechanism of Semi-Flexible Pavement Mixture

**DOI:** 10.3390/ma14185277

**Published:** 2021-09-14

**Authors:** Shiqi Wang, Huanyun Zhou, Xianhua Chen, Minghui Gong, Jinxiang Hong, Xincheng Shi

**Affiliations:** 1 School of Transportation, Southeast University, Nanjing 211189, China; zhyjy@seu.edu.cn (H.Z.); sxc1193918967@126.com (X.S.); 2 Jiangsu Sobute New Materials Co., Ltd., Nanjing 211103, China; gongminghui@cnjsjk.cn (M.G.); hongjinxiang@cnjsjk.cn (J.H.); 3 State Key Laboratory of High Performance Civil Engineering Materials, Nanjing 211103, China

**Keywords:** semi-flexible pavement, fatigue resistance, cracking mechanism, fatigue prediction, fracture surface

## Abstract

Semi-flexible pavement (SFP) is widely used in recent years because of its good rutting resistance, but it is easy to crack under traffic loads. A large number of studies are aimed at improving its crack resistance. However, the understanding of its fatigue resistance and fatigue-cracking mechanism is limited. Therefore, the semi-circular bending (SCB) fatigue test is used to evaluate the fatigue resistance of the SFP mixture. SCB fatigue tests under different temperature values and stress ratio were used to characterize the fatigue life of the SFP mixture, and its laboratory fatigue prediction model was established. The distribution of various phases of the SFP mixture in the fracture surface was analyzed by digital image processing technology, and its fatigue cracking mechanism was analyzed. The results show that the SFP mixture has better fatigue resistance under low temperature and low stress ratio, while its fatigue resistance under other environmental and load conditions is worse than that of asphalt mixture. The main reason for the poor fatigue resistance of the SFP mixture is the poor deformation capacity and low strength of grouting materials. Furthermore, the performance difference between grouting material and the asphalt binder is large, which leads to the difference of fatigue cracking mechanism of the SFP mixture under different conditions. Under the fatigue load, the weak position of the SFP mixture at a low temperature is asphalt binder and its interface with other materials, while at medium and high temperatures, the weak position of the SFP mixture is inside the grouting material. The research provides a basis for the calculation of the service life of the SFP structure, provides a reference for the improvement direction of the SFP mixture composition and internal structure.

## 1. Introduction

Semi-flexible pavement is a new type of pavement structure formed by replacing one or more layers of the traditional asphalt pavement surface with a semi-flexible composite material formed by pouring cement grouting material with porous asphalt mixture. It has been highly valued in road engineering in recent years. The annual application area of SFP in China is close to 1 million square meters. The application of SFP covers Jiangsu, Shanghai, Guangdong and other provinces [1,2,3]. SFP mixture is a composite pavement material formed by pouring specific cement slurry into macroporous asphalt mixture, which is named because its stiffness is between asphalt concrete and cement concrete [4]. Laboratory tests and engineering practices show that the SFP mixture have excellent shear strength, rutting resistance, high temperature stability [5,6], better durability than traditional asphalt pavement [7], and may help with mitigating urban heat islands in city centres [8,9]. Although SFP shows excellent rutting resistance and comprehensive road performance, SFP in many sections has different degrees of cracking, and a few sections have premature pavement failure due to rapid crack development [10,11].

By finite element calculation, it is found that the maximum stress in the structure does not reach the ultimate stress that SFP can bear, so the influence of fatigue on cracking needs to be considered. In order to improve the material and structure for crack resistance, it is necessary to judge the weak position of the SFP mixture and the crack control stress. In order to find the weak parts, Ding built a SFP finite element model through digital image processing technology, characterized the distribution of materials through three-phase material structure, inferred the weak points of materials in cracks through tensile strain, and considered that the probability of cracks appearing at the interface between asphalt and grouting materials was greater [12]. A. Setyawan believes that the strength of SFP mainly depends on the strength of cement binder, and the compressive strength of cold mix grouting composite is lower than that of hot mix grouting material [13]. For the judgment of control stress, Cai applied acoustic emission technology to detect the failure process of the SFP and its porous asphalt mixture in uniaxial compression test [14]. RA (quotient of rise time divided by amplitude) value and energy distribution show that the number of shear cracks of the SFP increases during compression [14].

For the improvement of structure, Chen believes that the cracks of the SFP are mainly reflection cracks [15]. The structural model of the SFP mixture is established through finite element method, and the shear force is the main factor for crack development by applying the principle of fracture mechanics and judging through the stress intensity factor [15]. Temperature also has an important influence on the crack resistance of the SFP mixture [16,17]. Based on the above research, the methods to improve the crack resistance of the SFP include: adding materials to strengthen the bonding between three-phase material interfaces [14], reinforcing three-phase materials [18], adjusting gradation or material mix ratio [19], optimizing pavement structure and layer position [20,21,22,23].

Phenomenological method is a more traditional fatigue performance research method. It is considered that fatigue is a phenomenon caused by strength attenuation under repeated action. There are many kinds of laboratory tests used in phenomenological method, mainly including splitting fatigue test, four-point bending fatigue test, semicircular bending fatigue test, etc. The local deformation of splitting fatigue test is large, and it is difficult to control the strain [24]. The loading mode of the four-point bending method is closer to the vehicle load of the actual pavement. The four-point bending operation is simple, and the theoretical cracking point expands into a region, which is suitable for uneven materials [25,26]. The SCB fatigue test can establish a fatigue prediction model with good correlation and high accuracy [27]. The specimen of SCB fatigue test has a notch, which is more suitable for the structure with initial crack. The test specimen is easy to make, the test process is easy to control, and the result parameters are stable. It can be compared and verified in combination with the experimental results of indoor preparation and field sampling samples, which is of great significance for engineering practical detection and indoor experimental research [28].

For the cracking phenomenon of SFP, there is little research on the mechanism of fatigue failure starting from fatigue cracking, and the calculation method of fatigue performance has not formed a system. It is often considered that the weak point of the SFP mixture is the interface between asphalt binder and other materials, while the impact on the integrity of the material caused by the brittleness of grouting material and the property difference between it and asphalt mixture is ignored.

In order to understand the fatigue performance and cracking mechanism of SFP and provide reference for the material and structural design of SFP, the specific objectives of this study are as follows:

1. Evaluate the fatigue resistance of the SFP mixtures under different conditions by SCB fatigue test. Establish fatigue prediction model of the SFP mixtures.
2. Analyze the composition of fracture surface in SCB fatigue test by digital image processing technology to investigate its cracking mechanism. Discuss the influence of the difference between asphalt binder and grouting material on the fatigue cracking mechanism under different conditions.

## 2. Materials and Methods

### 2.1. Materials

SFP is prepared by pouring cement grouting material into porous asphalt (PA) mixture. The PA mixture is composed of asphalt binder, aggregate, lignin fiber, and MA-100(Modified Agent-100) modifier. The cement grouting material was provided by Sobute New Materials Co. Ltd. (Nanjing, China).

Styrene–butadiene–styrene (SBS) modified asphalt was used as the basic asphalt binder. Its main technical indicators are shown in Table 1, and they all meet the technical requirements. Basalt was chosen as aggregate in this study and the gradation of PA is referred to Gong [29]. MA-100 modifier is a kind of asphalt interface reinforcing agent produced by Sobute New Materials Co. Ltd., and its dosage is determined by the weight of asphalt binder. Lignin fiber was included as the stabilizer of the mixture, and its dosage is determined by the weight of PA mixture. The optimal asphalt content was calculated according to the contribution from asphalt binder and modifiers and determined by Cantabro and drainage tests. The material composition and quality of PA mixture is shown in Table 2.

JGM^®^-301 grouting material is adopted in this study. Its main properties are shown in Table 3. The water cement ratio is 0.34. It is a kind of early strength cement, which only needs curing for 14 days in the standard curing room.

The preparation process of the SFP specimen for the experiment is as follows. Firstly, the cylindrical specimens of PA mixture is prepared using shear gyratory compactor (CONTROLS, Italy), and were sealed by tape after cooling to room temperature. The grouting material is poured into PA mixture, and the specimens after grouting are cured for 14 days in the environment with temperature of 25 °C and humidity of 90%. After curing, the specimens were cut into semicircles. The thickness and diameter of the specimens were 50 mm and 150 mm, respectively.

### 2.2. Experiment

The semicircular specimens were used for monotonic SCB test and SCB fatigue test [30,31]. Universal testing machines (IPC, Australia) were used to load the specimens. The specimens were slit with the target notch depth of 15 mm and width of 1 mm for the SCB fatigue test. In this paper, the fracture of the specimen was taken as the fatigue failure criterion, so the stress control method is adopted in the SCB fatigue test. Temperature and stress ratio were selected as the variables of the test and the test under each condition was repeated three times to reduce the error. The tensile strength of the SFP at different temperature values was obtained by monotonic SCB test. The calculation method of tensile strength is as follows [32]:

(1) $$\sigma_{t}=\frac{4.976F}{BD}\;$$

$\sigma_{t}$ is tensile strength, MPa; F is the value of peak load, N; B is the thickness of the specimen, mm; D is the diameter of the specimen, mm.

The tensile strength of the SFP is the average of three parallel tests. The fatigue load of different temperature values and stress ratio tests is calculated by tensile strength. The loading frequency of the fatigue test was 10 Hz. The scheme of SCB fatigue test is shown in Table 4.

### 2.3. Analysis Methodology

#### 2.3.1. Fatigue Prediction Model of Materials in Laboratory

The establishment of the SFP fatigue prediction model draws lessons from the establishment method of asphalt mixture fatigue prediction model, such as the SHRP model, Asphalt Institute (AI model), shell model, and the multivariable and multi parameter fatigue prediction model established by Tongji University and South China University of Technology. The main idea of fatigue prediction model is to establish the relationship between material stress or strain and fatigue action times, introduce the influence of temperature, consider the modulus of asphalt mixture, asphalt aggregate ratio and other factors to improve it. Based on the data of SCB fatigue test, a fatigue prediction model suitable for SFP can be established. The variables of the fatigue prediction model mainly include the stress of the material, the dynamic modulus of the SFP mixture, and the ambient temperature. Referring to the fatigue prediction model of asphalt mixture mentioned above, the stress and modulus are the base in the formula, and the other influencing factors are constant parameters and the index of e (base of natural logarithm). The basic form of the final SFP mixture fatigue prediction model is as follows:

(2) $$N_{f}=ke^{k_{1}\left(N-T\right)}\sigma^{k_{2}\left(N-T\right)+k_{3}}{\left|E_{0}\right|}^{k_{4}}$$

$N_{f}$ is fatigue life, times; T is temperature of test piece and environment, °C; σ is tensile stress, MPa; $\left|E_{0}\right|$ is dynamic modulus at 20 °C, MPa. N, $k_{1}$, $k_{2}$, $k_{3}$ and $k_{4}$ can be obtained by fitting the test results. In this study, only one mix proportion of the SFP is adopted, so $k_{4}$ is taken as 0.

The fatigue prediction model can be used to predict the fatigue life of materials in the laboratory and can be used to calculate the fatigue life of structures in engineering after modification.

#### 2.3.2. Analysis Method of Fracture Surface in SCB Fatigue Test

**Figure 1:**
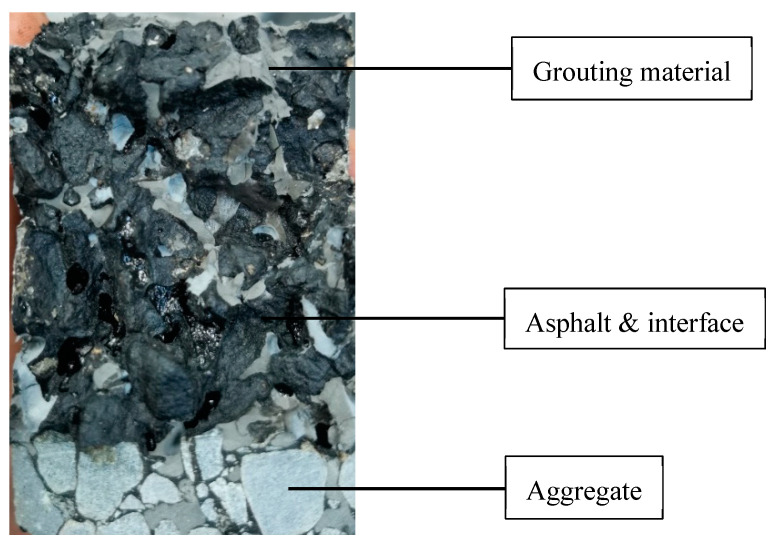
Fracture surface of the SCB fatigue test.

As shown in Figure 1 the fracture surface of the SFP mixture consists of aggregate, grouting material, asphalt and its interface with other materials (hereinafter referred to as asphalt phase). The color of aggregate and grouting material is gray white, which is quite different from that of asphalt phase, and the asphalt phase is pure black. The color of aggregate and grouting material is similar, so it is difficult to distinguish them in digital image processing. However, the aggregate is mainly distributed in the cutting seam in the cross-section, and hardly distributed in the fracture surface, which can be ignored in image processing. Therefore, it is only necessary to distinguish the two colors in the image to achieve the purpose of statistical distribution of grouting material and the asphalt phase in the fracture surface.

The original image was rotated and cut to get the fracture surface area, then it was binary processed by MATLAB [33]. Black pixels and white pixels represent the asphalt phase and grouting material, respectively, and their proportion in the total image pixels was calculated. The calculated results can be used to analyze the similarities and differences of the SFP fatigue failure process under different conditions, so as to further analyze the fatigue failure mechanism of the SFP.

**Figure 2:**
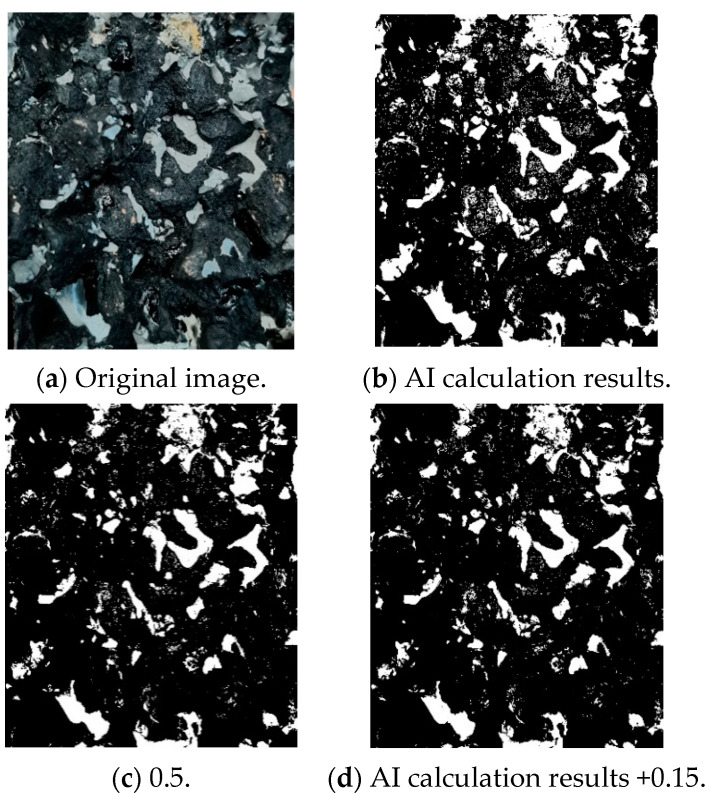
Selection of image binarization threshold.

The fracture surface image was randomly selected to determine the binarization threshold. As shown in Figure 2, the best effect was achieved by selecting the calculation result of gray thresh function of MATLAB plus 0.15 as the threshold. This threshold calculation method can reduce the errors caused by material reflection, camera aperture size, exposure time and so on.

## 3. Results and Discussion

### 3.1. SCB Fatigue Test Results

**Figure 3:**
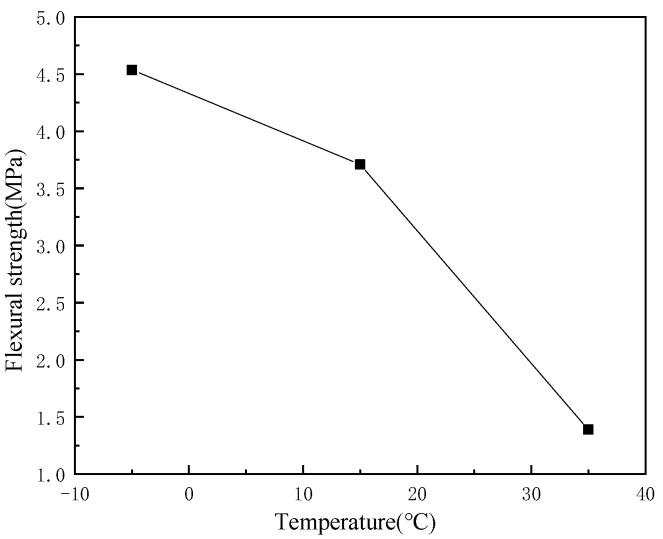
Flexural strength of the SFP.

**Figure 4:**
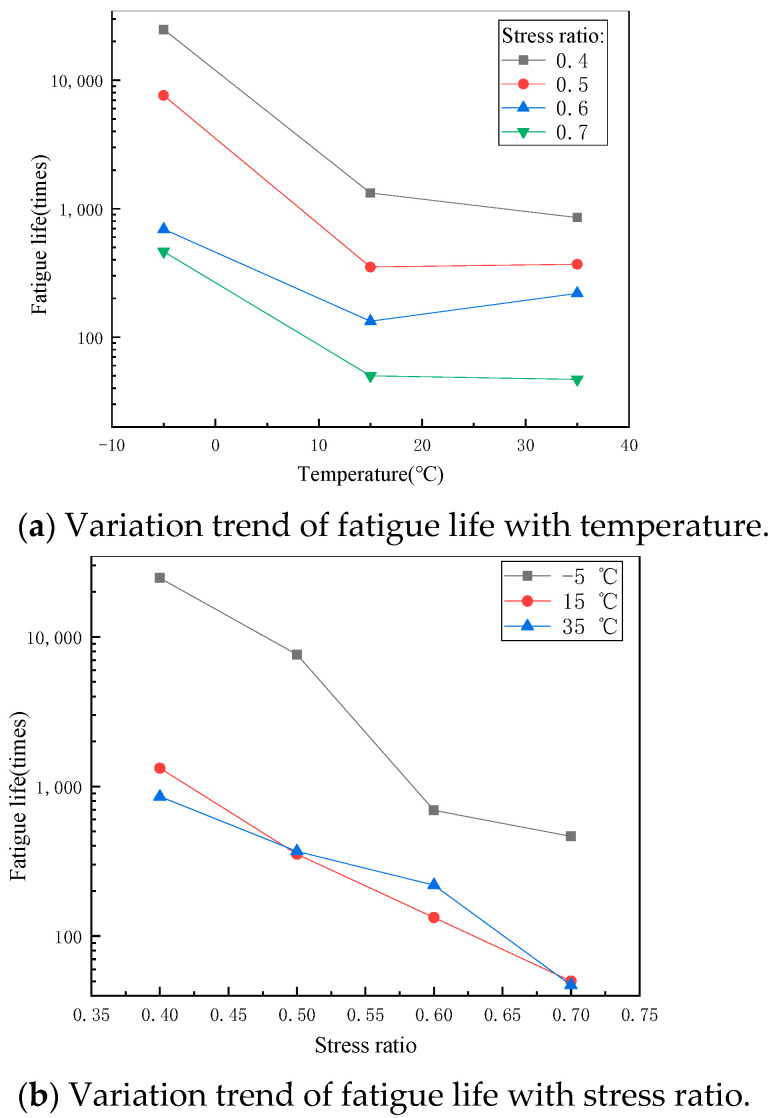
Fatigue life of the SFP mixture.

**Figure 5:**
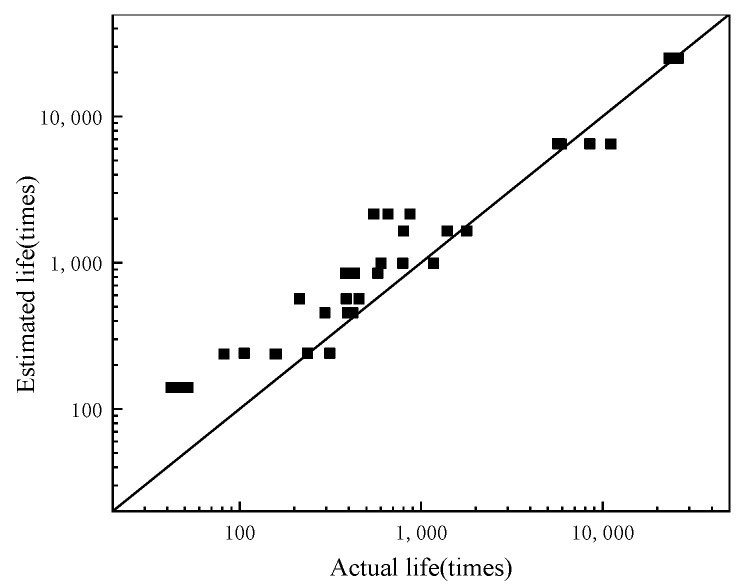
Comparison between estimated fatigue life and actual fatigue life.

The tensile strength of the SFP at different temperature values are shown in Figure 3, which is used as the basis for fatigue test. The variation trend of fatigue life with temperature and stress ratio is shown in Figure 4. Obviously, the properties of the SFP mixture are greatly affected by temperature and stress ratio: The flexural tensile strength decreases with the increase of temperature. When the stress ratio is low, the fatigue life decreases with the increase of temperature, and when the stress ratio is high, the fatigue life first decreases and then increases with the increase of temperature. The fatigue life decreases with the increase of stress ratio. The fitting results of the SFP fatigue prediction model are shown in Table 5. The fatigue prediction model R^2^ = 0.9937, and the fitting results are good. Compare the fatigue life prediction value calculated by the fatigue prediction model with the actual value obtained from the test, as shown in Figure 5. The analysis of Figure 5 shows that the predicted value of fatigue data is slightly larger than the actual value, but the change trend of the fatigue prediction model is the same. The expression can predict the times of material fatigue failure and judge the change trend of fatigue life under different conditions, which has reference significance in the design of semi-flexible pavement structure.

### 3.2. Discussion on Fatigue Performance of the SFP Mixture

**Figure 6:**
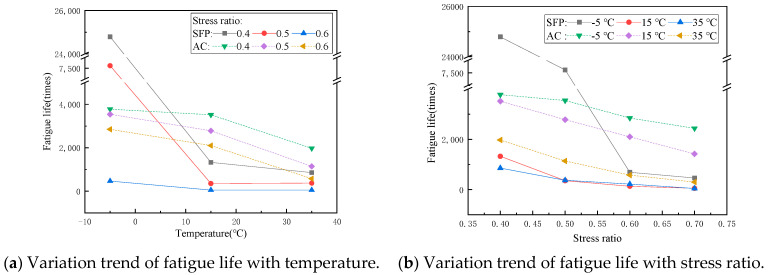
Fatigue life comparison of AC and SFP.

Taking the common asphalt mixture AC as the control group, the fatigue performance was measured by using the same test scheme as the above, which was used to evaluate the fatigue resistance of the SFP. Figure 6 shows the fatigue life comparison of the two materials under different environments. The fatigue properties of the two materials are significantly different, and the fatigue life of the SFP is much more sensitive to temperature and stress ratio. At low temperature and a low stress ratio, the fatigue life of the SFP is 4–5 times that of AC, which shows excellent fatigue resistance of the SFP. However, at a low temperature and high stress ratio, the fatigue life of the SFP decreases sharply, which has been significantly less than that of AC under the same conditions. At medium and high temperature, the fatigue life of the SFP is much less than that of AC regardless of the stress ratio. At the same stress ratio, from low temperature to medium temperature, the fatigue life of the SFP is greatly affected by temperature, while from medium temperature to high temperature is less affected by temperature.

According to all conditions, the SFP mixture can only have better fatigue resistance under low temperature and low stress ratio, and the fatigue resistance under other conditions is worse than that of asphalt mixture, and the fatigue resistance under high temperature and high stress ratio is far worse than that of asphalt mixture. This is determined by the properties of its constituent materials. The asphalt aggregate ratio of the parent asphalt mixture of the SFP mixture is similar to that of AC mixture used for comparison (4.2% and 5% respectively), but the asphalt binder content of the SFP mixture decreases after grouting. Based on the preliminary analysis of the characteristics of the SFP mixture and the above results, it is considered that the poor fatigue resistance of the SFP mixture is due to the low strength and poor deformation capacity of grouting materials. In the process of fatigue loading, it can be considered that microcracks of different sizes and numbers will be produced in the three materials. The generation time of microcracks and the number and size of microcracks depend on the crack resistance of the material itself. Asphalt has the best deformation ability in the three materials and has self-healing ability. The aggregate has high strength and good crack resistance, it is difficult to produce microcracks, and the fracture energy required for crack propagation is large. The early strength cement used for grouting material has less strength than aggregate, high brittleness, less deformation resistance than asphalt binder, so its crack resistance is the worst. Based on the characteristics of the three materials, the grouting material is most prone to brittle failure during fatigue loading, resulting in stress concentration at the crack tip, which makes the crack expand rapidly in the specimen. In conclusion, the grouting material is easy to crack, resulting in a concentration of stress, which makes the anti-fatigue performance of the SFP poor.

### 3.3. Digital Image Processing Results

**Figure 7:**
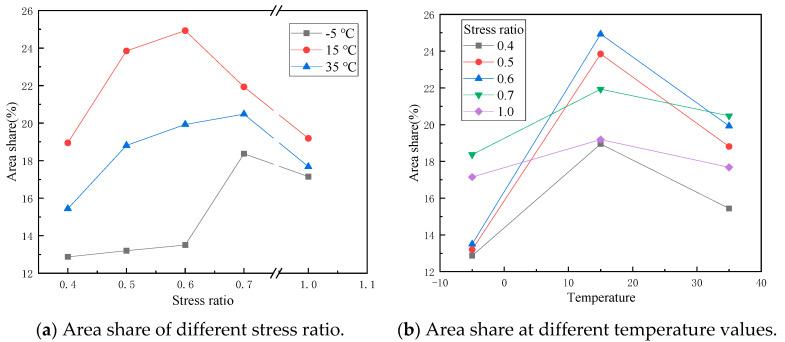
Area share of grouting material in the fracture surface.

Count the area share of grouting material in the crack surface of the specimen and take the average value of three parallel specimens as the final result. The trend of the area share of grouting material in the fracture surface with temperature and stress ratio is plotted as shown in Figure 7. Determined by Archimedes method, the connected porosity of parent macroporous asphalt mixture of the SFP mixture in this study is about 18%. Therefore, in the section of the specimen, the area of grouting material should account for about 18%. If the area of grouting material in the fracture surface is relatively small, it can be considered that the crack tends to bypass the grouting material when it occurs and expands. If the area of grouting material accounts for a large proportion, it can be considered that cracks tend to pass through the grouting material when they occur and expand. The analysis of Figure 7 shows that the area of grouting material in the fracture surface of SCB fatigue test accounts for 10–25%.

### 3.4. Discussion on Cracking Mechanism of the SFP Mixture

According to Figure 7a, when the stress ratio is less than 0.7, the area proportion of grouting material increases with the increase of stress ratio. Under a fatigue load, with the increase of stress ratio, the grouting material gradually becomes easier to crack than the asphalt phase. The strength of grouting material is higher than that of the asphalt phase. At a low stress ratio, the load has exceeded the fatigue failure stress of asphalt phase. Fatigue cracks begin from the asphalt phase and expand to the whole specimen with the asphalt phase as the main path. Early cracks will also appear in a few grouting materials at stress concentration positions. When the stress ratio increases gradually, the stress of grouting material gradually reaches and exceeds its fatigue failure stress. Due to the large brittleness and poor deformation capacity of the grouting material, the cracks occur earlier than the asphalt phase, and the propagation path of the cracks includes the asphalt phase and the grouting material. When the stress ratio is greater than 0.7, the area proportion of grouting material is close to 18%, which is due to less fatigue times under high stress ratio, and the failure form of material is more similar to single loading failure.

According to Figure 7b, the proportion of grouting material in the area of fracture surface first increases and then decreases with the increase of temperature, which may be due to the fact that the temperature sensitivity of cement grouting material is not as good as that of asphalt mixture. At low temperatures, the stiffness of the asphalt phase and the cement grouting material are both large, and close to brittle failure. However, because the strength of asphalt is lower than that of cement grouting material, its failure time is earlier when the temperature and stress are relatively low, so the area of grouting material in the fracture surface is relatively small. At medium temperature, the strength of asphalt phase and grouting material decreases. Asphalt has higher temperature sensitivity. With the increase of temperature, its deformation capacity improves, and the grouting material is still brittle failure. At the same time, due to the faster decline of modulus of asphalt phase, the grouting material shares a greater proportion of stress, and the cracking time of grouting material is earlier than that of asphalt phase, so the area proportion in the fracture surface increases. At a high temperature, the strength of asphalt decreases obviously, and the proportion of stress shared by grouting material continues to increase after asphalt softening. However, because the decrease of asphalt strength exceeds the increase of stress borne by grouting material, the area proportion of asphalt phase rises somewhat.

In actual use, due to the high strength of aggregate, the loading stress is difficult to cause damage. Asphalt has a self-healing ability.

During fatigue loading, the process of microcrack generation, incomplete self-healing, crack generation and propagation is repeated in the asphalt phase until the material is completely destroyed. The asphalt phase is the main factor controlling the fatigue resistance of asphalt mixture. The difference between the SFP mixture and asphalt mixtures lies in the role of grouting material in the destruction process. The strength of the grouting material is less than that of the aggregate. During the fatigue loading process, microcracks continue to occur in the grouting material, but the grouting material does not have the self-healing ability. At the same time, the stress concentration at the crack tip weakens the self-healing ability of the asphalt phase, and finally leads to the acceleration of the overall failure process of the specimen.

In conclusion, the difference of material properties and temperature sensitivity between the asphalt phase and the grouting material leads to the difference of fracture surface composition of specimens under different conditions. This phenomenon reveals that the poor fatigue resistance of the SFP mixture at medium and high temperature is caused by the brittleness of the grouting material, the difference of modulus between the grouting material and the asphalt, and stress concentration.

## 4. Conclusions

The fatigue resistance of the SFP mixture under different temperature values and stress ratio was evaluated by the SCB fatigue test, and the laboratory fatigue prediction model of the SFP mixture is established. The fatigue cracking mechanism of the SFP mixture is analyzed by a digital image processing technology. A fatigue prediction model for SFP structure calculation is derived. The conclusions are as follows:

1. At a low temperature and low stress ratio, the fatigue resistance of the SFP is 2–7 times that of AC. At a medium temperature or high stress, the fatigue resistance of the SFP suddenly drops to 15–45% of AC. Under the condition of high temperature and a high stress ratio, SFP almost loses its anti-fatigue ability.
2. The main reason for the poor fatigue resistance of the SFP mixture is the poor deformation capacity and low strength of grouting materials.
3. The performance difference between grouting material and asphalt binder is large, which leads to the difference of fatigue cracking mechanism of the SFP mixture under different conditions.
4. Under a fatigue load, the weak position of the SFP mixture at la ow temperature is asphalt binder and its interface with other materials, while at medium and high temperatures, the weak position of the SFP mixture is inside the grouting material.
5. The research on improving the fatigue resistance of the SFP mixture at medium and high temperatures can start from improving the deformation resistance and strength of grouting material.

This study points out that fatigue cracking is one of the main forms of the SFP structural cracking, and proposes a fatigue prediction model for SFP mixtures to provide a reference for structural design and life calculation, and provides research directions for improving the crack resistance of the SFP mixtures from the aspects of material composition and material modification.

There are two main limitations of this study. First, only one mix proportion of the SFP mixture is selected, and the fatigue prediction model has room for further optimization. Secondly, the cracking mechanism of the SFP mixture is analyzed from the surface after fracture, and the observation of fatigue cracking process is lack. In the later research, it is suggested to adjust the mix proportion of the SFP mixture to improve the fatigue prediction model of the SFP. It will be very effective to monitor and analyze the entire process of fatigue cracking of the SFP mixture with the help of real-time computer tomography and digital image correlation technology.

## Figures and Tables

**Table 1:** Main technical indicators of SBS modified asphalt.

Properties	Unit	Test Value	Requirements (GB T 494–2010)
Softening point	°C	75	≥60
Ductility (5 °C)	cm	30	≥20
Penetration (100 g, 25 °C)	0.1 mm	54	40–60
Storage stability	°C	1.2	≤2.5
Viscosity	Pa·s	2.0	≤3.0
Flash point	°C	311	≥230
Elasticity recovery (25 °C)	%	92	≥75

**Table 2:** Material composition and quality of PA mixture.

Mixture Component	Content
Aggregate (mm)	98.0
Limestone powder	2.0
SBS Modified asphalt	4.2
MA100	0.3
Lignin fiber	0.2

**Table 3:** Main properties of grouting material.

Fluidity/s	Setting Time/h		Compressive Strength/MPa					24 h Bleeding Rate (%)	Dry Shrinkage Ratio (%)
Initial	30 min	Initial	Final	2 h	3 h	3 d	28 d		
10.3	12.6	0.9	1.4	15.8	24.0	34.5	42.3	0.1	0.14

**Table 4:** Scheme of SCB fatigue test.

Factor	Content
Material	SFP-13
Stress ratio	0.4, 0.5, 0.6, 0.7
Temperature (°C)	−5, 15, 35
Load frequency (Hz)	10
Times of parallel tests	3

**Table 5:** Fitting results of fatigue prediction model.

k	$k_{1}$	$k_{2}$	$k_{3}$	N	R^2^
81.746	0.222	0.0638	−3.371	36.986	0.9937

## Data Availability

Data is contained within the article.
